# The Impact of COVID-19 and Associated Restrictions on Physical Activity Among Assisted Living Residents

**DOI:** 10.1093/geroni/igab046.1614

**Published:** 2021-12-17

**Authors:** Elizabeth Galik, Barbara Resnick, Rachel McPherson

**Affiliations:** 1 University of Maryland School of Nursing, Baltimore, Maryland, United States; 2 University of Maryland Baltimore and Baltimore County, Catonsvile, Maryland, United States

## Abstract

COVID-19 and associated restrictions significantly impacted residents in assisted living (AL) communities. This was a descriptive study of 35 AL communities that were participating in an implementation trial of Function Focused Care for Assisted Living Residents with Dementia during the COVID-19 pandemic. Within twelve months of the COVID-19 pandemic, 18% of the AL communities had at least one resident who was positive for COVID-19. Almost half of the ALs allowed health care providers into the setting. All of the ALs facilitated family visits outside and by telephone and technology, but only 11% allowed visitors inside the community. Over 50% stopped using recreational supplies to encourage physical activity and 28% reported that residents experienced more behavioral and psychological symptoms of dementia. Restrictions designed to prevent the spread of COVID-19 may have negatively impacted resident behavior and the AL staff’s engagement of residents in physical and recreational activities during the pandemic.

